# Editorial: Prolonged grief disorder: vulnerability and resilience

**DOI:** 10.3389/fpsyt.2024.1387937

**Published:** 2024-02-27

**Authors:** Mushtaq Margoob, Mudasir Firdosi, Mohammad Zia Ul Haq Katshu

**Affiliations:** ^1^ Advanced Institute of Management of Stress and Life Style Related Problems, Srinagar, Jammu and Kashmir, India; ^2^ Supporting Always Wholeheartedly All Broken−Hearted, Kashmir (SAWAB), Voluntary Medicare Society, Bemina, India; ^3^ Kent and Medway NHS and Social Care Partnership Trust, Canterbury, United Kingdom; ^4^ Kent and Medway Medical School (KMMS), Canterbury, United Kingdom; ^5^ Institute of Mental Health, School of Medicine, University of Nottingham, Nottingham, United Kingdom; ^6^ Nottinghamshire Healthcare NHS Foundation Trust, Nottingham, United Kingdom

**Keywords:** grief, prolonged grief disorder (PGD), depression, trauma, life events

Separating normal from abnormal human experience lies at the heart of medicine. And in no other field of medicine has this been so contested as in Psychiatry. Grief, following the death of a loved one, is universal, but the duration, expression, and impact of grief on the bereaved varies substantially within and across cultures. Unsurprisingly, differentiating normal from abnormal grief has proved controversial. Various concepts have been proposed over the years to define pathological or unusual grief responses, including complicated grief disorder (1), prolonged grief disorder (2) [PGD; e.g. Prigerson et al. (2)], complicated grief (3) [e.g. Shear et al. (3)], and persistent complex bereavement disorder (4) (American Psychiatric Association, 2013). These proposals have finally resulted in the formal inclusion of prolonged grief disorder (PGD) in the 11th edition of the International Classification of Diseases (ICD-11) (5) and the Text Revision 5th Edition of the Diagnostic and Statistical Manual of Mental Disorders (DSM-5-TR) (6). This, on the one hand, is a reflection of global recognition of the clinical relevance of grief-related disorders and will encourage mental health professionals to help and support those who need help following bereavement (7). On the other hand, it has reignited the debates about the concept of pathological grief, its phenomenological overlap/distinction from depression, cultural biases in the current diagnostic criteria, and the medicalisation of the human experience. It also runs contrary to the move away from categorical diagnosis to symptom dimensions in psychiatry, which has gained substantial momentum based on evidence from genomic, epidemiological, phenomenological, neuroimaging, and course and prognosis studies. This theme tries to capture the uncertainties related to conceptualisation, classification, and transcultural aspects of abnormal grief as well as potentially relevant risk factors and resilience correlates for Prolonged Grief Disorder (PGD) among children.

Gouveia provides a brief overview of the universality of the grieving process from an evolutionary perspective and how cultural influences shape its expression. Gouveia has brought into sharp focus the controversies around defining pathological grief and differentiating it from depression. The author has rightly pointed out that as elaborated by Henrich et al. (8), most of the evidence to define pathological grief comes from Western, Educated, Industrialised, Rich, and Democratic (W.E.I.R.D.) participants, posing serious questions on its universal application. They suggest the inclusion of views from different cultural backgrounds as well as related disciplines, including anthropology, sociology, and ethnography, to arrive at a universally applicable definition of pathological grief. They acknowledge that while we strive for this ambitious plan, we need to leave our entrenched positions and ensure that people who have suffered bereavement and need help from mental health professionals receive the support they need.

Treml et al. estimated the point prevalence of PGD from a German population sample to assess the diagnostic agreement between ICD-11 and DSM-5-TR. There was substantial agreement between the two classification systems (k=0.75; 95% CI 0.66 – 0.85). Notably, the diagnostic agreement increased by increasing the threshold for diagnosis in ICD-11. However, there was a significant difference in the point prevalence with higher estimates using ICD-11 (6.8%) compared to DSM-5-TR (4.7%). Their work highlights the need for harmonising the definition and criteria for diagnosis of PGD across diagnostic systems (9).

Frei-Landau proposes a model based on the teacher-school mental health professional dyad to support bereaved children. The proposed model is based on Winnicott’s (10) parental dyad, wherein the mother’s role is to provide the physical and emotional space to enable the child to feel safe while the father’s role is to provide a safe environment for the mother to enable her to fulfil her role. Frie-Landau’s proposal is interesting and needs empirical evidence to support it.

The bibliometric analysis of grief interventions (GI) by Li et al. shows an exponential increase in the number of publications in this area over the last two decades. They show that a large proportion of the research work in GI has been done in North American and European populations, understandably by researchers based in Universities from the same regions, and published in journals from the same region. Their work brings into sharp focus the widely held belief that most of the research in this area is limited to a particular cultural context and therefore lacks generalisability.

The incorporation of PGD in ICD-11 and DSM-5-TR has rejuvenated interest in understanding and conceptualising grief and rekindled the controversies in teasing apart normal from abnormal grief. Future work in this area should incorporate the fundamental changes in our understanding of mental illnesses from the current categorical classification to those based on marrying phenomenological descriptions with underlying aetiological and pathophysiological mechanisms.

## Author contributions

MM: Conceptualization, Writing – original draft, Writing – review & editing. MF: Writing – original draft, Writing – review & editing, Conceptualization. MK: Writing – original draft, Writing – review & editing, Conceptualization.
